# Consumers acceptability of using screen capture methods to capture marketing strategies on online food delivery platforms: a qualitative study

**DOI:** 10.1017/S1368980025000515

**Published:** 2025-04-14

**Authors:** Adyya Gupta, Kathryn Backholer, Catherine E Huggins, Gloria KW Leung, Rebecca Bennett, Anna Peeters

**Affiliations:** Deakin University, Institute for Health Transformation, Global Centre for Preventive Health and Nutrition, School of Health and Social Development, Faculty of Health, Geelong, VIC 3220, Australia

**Keywords:** Online food delivery, Qualitative methods, Digital marketing strategies, Screen capture method, Digital food environment

## Abstract

**Objective:**

The use of online food delivery (OFD) platforms is on the rise, and currently, there are no public health policies that regulate what and how food is sold on these platforms. Research quantifying and describing the marketing strategies on OFD platforms is limited. Our study aimed to test the consumers’ acceptability of using two screen capture methods to record their food purchasing behaviour on OFD platforms and describe consumers’ exposure to, and engagement with, marketing strategies on OFD platforms in real time.

**Design:**

Semi-structured online interviews on the consumer acceptability of using the screen capture methods were analysed using thematic analysis. Screen recordings of OFD orders were analysed using content analysis, guided by the marketing mix framework (i.e. product, placement, price and promotion).

**Settings:**

Victoria, Australia

**Participants:**

Twenty adults using the OFD service at least once a month were recruited.

**Results:**

The mean age of the sample was 28 years. 75 % were females, over 80 % had completed higher education and 20 % lived with children < 18 years of age. Over half used OFD service two to five times per week. Participants expressed that both smartphone’s in-built screen recording function and third-party screen recording application are easy-to-use and time-efficient with high levels of user satisfaction. A range of marketing strategies were observed on the OFD platform. These included the presence of, and strategic placement of selected food products, price discounts and promotion strategies. Participants appeared to engage with marketing strategies through multiple clicks to reduce the total cost of their OFD order.

**Conclusions:**

Our study demonstrates that screen recording is an acceptable method for capturing and assessing consumers’ real-time exposure to, and engagement with, a range of marketing strategies on the OFD platform. Studies with larger samples are needed to substantiate our findings.

A diet high in calories, sugars, salt and fat is a well-known risk factor for chronic health conditions including overweight, obesity, diabetes, CVD and some cancers^(1,2)^. Food environments are settings where food-related decisions are made^(3)^. Food environments play a vital role in determining population diets and health outcomes through the availability, accessibility and marketing of food options^(4)^. Online food delivery (OFD) platforms are a disruptive innovation within the food retail environment^(5)^ that is rapidly expanding and facilitating easy access to unhealthy calorie-dense food options. Their user-friendly interface makes the process of selecting and purchasing food and drinks simple^(6)^. This has resulted in the increased use and popularity of OFD platforms for purchasing out-of-home meals.^(7)^ This platform-to-consumer business model (e.g. Uber Eats^(8)^, Deliveroo^(9)^) electronically connects consumers to a broad range of food service outlets and is a substitute for the conventional forms of acquiring out-of-home meals^(7)^.

Whilst there is some evidence showing the widespread use of marketing strategies (for example, advertising, product placement and branding) by large transnational food brands on digital media such as websites and social media channels^(10–12)^, there is very little evidence on marketing strategies used on OFD platforms. As evidence in this field is still emerging, few studies^(13–16)^ have described marketing strategies, such as price discounts, combo deals and appealing food images to promote unhealthy, calorie-dense food items on OFD platforms. These studies indicate that OFD platforms use persuasive marketing strategies to enhance user experience, desirability and the intentions to purchase unhealthy food. However, they do not provide clear guidance on consumer’s actual purchase behaviour and the impact of immediate factors like persuasive promotions that may inform the actual food purchasing decisions. Actual (or real-time) food purchase behaviour data offers a more accurate and actionable insight into consumer behaviours compared with purchase intentions. Given the upsurge in the popularity and use of OFD platforms^(7)^, with evidence of the low nutritional quality of foods sold on the OFD platforms^(17–19)^, there is a need to understand how marketing techniques are used to influence consumer purchasing and consumption behaviour through OFD platforms on their platforms.

There are no studies to date that have quantified and described the nature and extent of marketing strategies present on OFD platforms. To monitor consumers’ exposure to, and engagement with, the dynamic food marketing strategies on online settings, the WHO has developed a CLICK monitoring framework^(20)^. The CLICK framework recommends using screen capture methods to obtain real-time data on consumers’ exposure to, and engagement with, online food marketing strategies. Some of the commonly used screen capture methods^(21,22)^ in health research include screen recording, wearable cameras, eye tracking devices and screenshots. Of these, only screen recordings can continuously capture what users are seeing on the screen of a digital device (such as a smartphone or computer) at any given time. All other methods are limited in their ability to continuously capture the dynamic online behaviour due to their static nature. For example, wearable cameras^(23)^ or eye tracking devices can only capture the user’s activity based on the duration of eye movement, and screenshots^(24)^ only provide static snapshots of the screen, limiting their ability to continuously track users’ activities. Methods such as think-aloud protocols^(25)^ are increasingly being used in technology research to capture verbal commentaries alongside other screen capture methods to gain insights into people’s experiences when interacting with new technologies. However, these techniques are often criticised for increasing user’s cognitive load as the users have to continuously transform thoughts into words in a quiet place while undertaking the task^(26)^. Screen recordings have been previously shown to be useful in the field of education to deliver high-quality teaching^(27)^. In recent times, screen recording has been utilised in market research to gather consumer data and tailor marketing strategies to enhance consumer experiences in the online environment^(28)^. Screen recordings can be undertaken using an inbuilt feature on a smartphone or via a third-party application. A small number of studies have reported using screen recording as a potential tool to study children and adolescents’ exposure to food and beverage marketing in social media apps^(29,30)^. The screen recordings in these studies showed a concerning volume of invasive marketing techniques used to promote unhealthy food options to children and adolescents. No study has utilised this methodology to investigate consumer behaviour on OFD platforms.

Furthermore, the evidence on the acceptability and usefulness of conducting screen recording while using OFD platforms remains unknown. Assessing acceptability is reported as an important consideration in the appraisal of interventions to inform the usability and engagement with the intervention^(31)^. The theoretical framework of acceptability is widely applied in health research to assess the intervention acceptability from the perspectives of people receiving or delivering the interventions^(31,32)^. In the context of our study, in addition to assessing the usability of screen recording methods, we also explored user’s feedback on the compatibility of using different screen recording methods with smartphone devices, and privacy and data storage capabilities. We argue that understanding the acceptability of screen capture tools will help gain insights into users’ exposure to and engagement with OFD platforms in real time and demonstrate the potential of using this methodology in future research investigating unhealthy food marketing on online platforms.

The aims of this study were to test the consumers’ acceptability of using two screen capture methods to record their food purchasing behaviour on OFD platforms and describe consumers’ exposure to, and engagement with, marketing strategies on OFD platforms in real time.

## Methods

A qualitative descriptive study was undertaken along with a content analysis to allow descriptions on a topic, of which little is known. Using a phenomenological qualitative approach^(33)^, we were able to explore how individuals interpret the screen capture method, through constructs such as usability, trust and perceived benefits. This further enabled us to understand the meanings individuals assign to their experiences, emphasising context (use of OFD platforms) constructed through social interactions and personal perspectives. The Standards for Reporting Qualitative Research were used in reporting this study^(34)^. The study was approved by Deakin University Human Research Ethics Committee (HEAG-H 37_2023).

### Theoretical perspective

The theoretical framework of acceptability^(31)^ provides a structured, evidence-based guide for assessing the acceptability of an intervention based on a range of cognitive and emotional responses from participants. Using a theoretical framework of acceptability^(31)^, we gathered participant’s experiences of using the two screen capture methods. We then identified and mapped the marketing strategies on OFD platforms as observed from the screen recordings of the online food order to the Marketing Mix framework^(35)^ (including product, placement, price and promotion).

### Sampling and recruitment

English-speaking adults aged 18–45 years, using OFD platforms at least once a month and living in Victoria, Australia were eligible to participate. This eligibility criteria were based on the previously reported sociodemographic characteristics of OFD platform users^(7,36,37)^. Participants were recruited through convenience sampling from July 2023 to September 2023. The study was advertised via university-led social media accounts (including Twitter, Facebook and LinkedIn). Participants who contacted the primary author and expressed interest to participate were emailed a copy of the plain language statement and were enrolled after providing written informed consent. Upon enrolment, participants were allocated to one of the two screen capture groups based on the order in which they were enrolled in the study, namely, using smartphone’s in-built screen recording function (hereon referred as ‘phone group’), and using a third-party screen recording app (hereon referred as ‘app group’). Sampling was concluded when an equal number of participants were recruited to each of the two screen capture groups through random allocation and when no new concepts emerged during the analysis.

### Data collection procedure

All participants were emailed an instruction sheet detailing the required steps, prior to starting the task. Participants were required to complete a screen capture task, i.e. screen record the process of placing one full OFD within a 2-week period using any OFD platforms and upload their screen recording data to the University’s secured server. The third-party app only allowed a recording up to 10 min in duration for upload, while the smartphone’s in-built function for screen recording did not have any duration restriction for the upload of the recording. To ensure no personal information was captured, prior to commencing the task, participants were instructed to turn off all notifications, not to switch between platforms or apps whilst recording, to blackout any sensitive information (e.g. banking details) through video editing functions or to permanently delete any screen capture data if personal information was captured and to restart the task, if required. The screen recordings for participants in the app group were uploaded automatically to a secure dashboard that was only accessible to the research team. Participants in the phone group were sent a unique link to upload their screen recording videos to a secure password-protected University database. In addition to the recordings, all participants were asked to upload a screenshot of their order receipt confirming the order, and a screenshot of their previous food order purchased over the last month using the same OFD platforms, to assess if the marketing strategies on OFD platforms were personalised or targeted based on previous food orders. A reminder email to complete the task was sent to participants 1 week after the commencement of the study period. The study was conducted between July and September 2023.

Participants were given 2 weeks to complete the screen capture task and within a week after the task completion, 30–45 min semi-structured in-depth online interviews were conducted. First, participants’ basic demographic and OFD platforms-related information were collected using a short survey. These details included age, sex, household income, highest education completed, postcode and number of children less than 18 years of age living in the household. OFD platforms-related information was also collected, including frequency of OFD platform use, choice of OFD service used and whether participants held a membership with the OFD platforms (which provides the members with discounts on delivery fees and other charges). Next, in-depth semi-structured online interviews were conducted with participants (online Supplementary Table 1). The purpose of the interview was to understand the acceptability of the screen capture methods used. This included participants’ perceptions of how they felt about the task and why, the extent to which they understood the task and if and how the study instructions helped them with performing the screen recording task. Probes were used to gather insights into the most challenging and/or useful aspect of the screen capture method. The interviews were conducted by the primary author using the Zoom software platform between September and October 2023. At the end of the interview, participants received a supermarket voucher for 50 Australian Dollars ($AUD) (equivalent to United States Dollar ($USD) 33) as a thanks for participation. A paid transcription service was used to transcribe the audio-recorded interviews verbatim. To verify the transcripts for accuracy and reliability, the primary author self-transcribed 50 % of the interviews (*n* 10) and cross-checked them with the transcripts provided by the transcription service.

### Data analysis

All audio transcripts and screen recording data were imported into QSR-NVivo 12 software for coding and analysis. Each participant was assigned a unique code to track specific quotes from that individual within the data set to maintain anonymity. For example, B1 refers to the specific quote number for participants enrolled within the phone group, while C1 refers to the specific quote number for participants enrolled within the app group. Using thematic analysis^(38)^, a two-step coding process was undertaken by the primary author whereby first, the audio transcripts were read and coded to identify words and phrases describing the acceptability of using multiple screen capture methods. The theoretical framework of acceptability^(31)^ was used to group the codes under the selected constructs of the framework, namely, affective attitude, burden, ethicality, perceived effectiveness and self-efficacy (Table 1). Next, the recurring codes within the constructs were reviewed by the team, and two themes were constructed to capture the acceptability and usefulness of using the screen capture methods for future research.

**Table 1.** Participant characteristics (*n* 20)

Participant characteristics	
Age (mean, range)	28 years (18–45 years)
Sex	
Male (%)	25 % (*n* 5)
Female (%)	75 % (*n* 15)
Education level (%)	
High school	15 % (*n* 3)
Bachelor’s degree	45 % (*n* 7)
Postgraduate degree	40 % (*n* 10)
Household income per annum (%)	
< $AUD 19,000 [USD 12,294]	5 % (*n* 1)
$AUD20,000 [USD12,940] to < $AUD49,0000 [USD31705]	5 % (*n* 1)
$AUD 50,000 [USD 32 352] to < $AUD79,000 [USD 51 115]	15 % (*n* 3)
>= $AUD80,000 [USD 51,762]	75 % (*n* 15)
Household status (%)	
Households with children < 18 years of age (%)	20 % (*n* 4)
Households with no children < 18 years of age (%)	80 % (*n* 16)

AUD, Australian dollars; USD, United States dollars; *n*= sample number.

Next, the screen capture data in the form of video recordings were analysed using content analysis, to describe consumers’ real-time exposure to, and engagement with, marketing strategies on the OFD platform. The screen recording data were first viewed to assess the time spent by the users on different OFD platforms to make an online food order and the total $AUD spent to purchase an OFD order. The second viewing focussed on identifying the commonly arising marketing strategies. These marketing strategies were then categorised under the traditional Marketing Mix framework using 4Ps- product, placement, price and promotion^(35)^. In the context of our study, product included product mix (i.e. the assortment of products viewed on the app or using search filters), placement included the appearance of recommended, best match, featured or popular tags on a range of food offerings and food outlets appearing throughout the purchase and in particular on the homepage (first page displayed) of the OFD platform, price included discounts, rewards, time-limited deals; promotion included the use of banner advertisements, push notifications and member special offers. Additionally, to identify whether food products that appeared on the participants OFD platform were based on their previous food orders, we manually matched the top five food products that appeared on the homepage, food outlet page and at checkout in the screen recordings to the screenshots of the history of previous food orders provided by the participants purchased using the same OFD platform. The data were grouped based on similar food products that appeared in the history of food orders and across all pages of the OFD platform. We then assessed the marketing strategies that were applied to these specific food products. This provided an indication of whether or not and to what extent the marketing strategies were personalised. Participant’s engagement with the marketing strategies was assessed based on their clicks to navigate through strategies to influence search, selection and purchase on the OFD platform. The coding was initially conducted by the primary author (approximately took less than 20 min to code one video) and then was reviewed and finalised by discussion with the team.

## Results

### Participant characteristics

A total of twenty eligible people who expressed interest to participate, completed the study, with ten each in the two screen capture groups. The mean age of the participants was 28 years (age range: 18–45 years), and 75 % of them were women (*n* 15). Participants mostly lived in metropolitan suburbs (classified as having a high socio-economic status) of Melbourne, Victoria, based on the Australian Bureau of Statistics Socioeconomic Indices for Areas (*n* 18). 75 % of the participants (*n* 15) earned more than $AUD80,000 (after tax) ($USD 51,762) and over 85 % (*n* 17) held bachelor’s or post-graduate degrees. Twenty percent of the households included one or more children less than 18 years of age (*n* 4).

Three leading OFD platforms in Australia were used by the participants namely, Uber Eats (*n* 15), DoorDash (*n* 3) and Menulog (*n* 2). As per the screen recordings of the OFD order, most participants (*n* 15) placed dinner orders over the weekdays. The total $AUD spent on OFD orders ranged from $AUD35 (USD23) to $AUD60 (USD39). Fifteen participants (75 %) self-reported that they used OFD platforms for food purchases 2–5 times per week. Five participants (25 %) held premium memberships with an OFD platform which enabled them to receive a discount on delivery fees and other ‘members only’ promotional discounts. The length of screen recordings uploaded by participants ranged from 2 to 5 min. Most participants (*n* 8 out of 10) within the phone group used Apple iOS to perform the screen recording task. No difference in the overall ability or the process of undertaking the screen recording task was reported by participants based on the smartphone brands. Table 1 shows the characteristics of all the study participants.

### Acceptability of using screen capture methods to capture food purchases on online food delivery service

Overall, the screen recording methods were acceptable to participants. Participants in both groups described their experience of using the screen recording methods as easy and time efficient, and they also described these methods as acceptable to capture their real-time exposure to, and engagement with, the marketing strategies of the OFD service (Table 2). Differences in participants’ feedback on the acceptability of using the two screen capture methods are described below under two broad themes, namely, user satisfaction and, barriers and facilitators to the use of screen capture methods (described as a combined theme).

**Table 2.** Theoretical framework of acceptability of using two screen recording methods to capture food purchase on OFD platforms (*n* 20 screen recordings)

Theoretical framework of acceptability domains	Definition	Themes generated	Quotes from the group using mobile in-built screen recording function	Participants (total *n* 10)		Quotes from group using a screen-recording app	Participants (total *n* 10)		
				*n*	%		*n*	%	
Affective attitude	How an individual feels about using the screen capture methods to undertake video recording of the food purchase on OFD platforms (‘the task’)	User-satisfaction	‘To be honest I did not find any difficult. I think it was very smooth.’ (B10, phone group)	9	90	‘Was quite happy with the whole experience.’ (C21, app group)	10		100
Intervention coherence	The extent to which the participant understands the screen capture methods and how it works	User-satisfaction	‘Yeah, very easy because I’ve used the screen recording feature before, so I was very familiar where it is and how do I record, where it gets saved. (B10, phone group)	10	100	‘I think it was a pretty easy process. It’s just screen recording and then you stop when you’re done so no issues with that.’ (C25, app group)	10		100
Self-efficacy	The participant’s confidence that they can perform the task required using the screen capture methods	User-satisfaction	‘I spent around – no more than two minutes because I’m very familiar with the recording thing.’ (B1, phone group)	10	100	‘Maybe 10–15 min. I was getting used to the app then. That’s the only things. Because of course it’s a new app, you have to get used to like an app before you proceed with the thing. Other than that, I didn’t take much of a time.’ (C10, app group)	7		70
Opportunity costs; Burden	The extent to which benefits, profits or values must be given up and perceived effort required when engaging with the screen capture methods to undertake the task	Barriers	‘There were a couple of times where I did - I would get a message or something while it was screen recording and either I’d just forget and I’d like open my messages, or it - I guess it was just kind of like privacy reasons’. (B3, phone group)	7	70	‘I wasn’t concerned, but I can see maybe some people might say, might be a little concerned about data protection, data privacy here. But there’s just a lot, as you know, in the media about the potential dangers of downloading apps’ (C14, app group)	8		80
Ethicality; Perceived effectiveness	The extent to which using the screen capture methods was a good fit with individual’s value system; perceived as likely to achieve its purpose i.e. to undertake the task	Facilitator	‘I think just the simplicity of the instructions and how clearly, they were laid out. It was good. I think it was very succinct.’ (B7, phone group)	10	100	‘Loved the fact that you have the instructions uploaded on the app itself, So I feel like the interface and the instructions were clear and it was very easy to us (C21, app group)	10		100

#### User-satisfaction

Nearly all the participants across both groups expressed a good understanding of how screen recording methods had to be applied to undertake the task. Participants expressed a high level of ease and familiarity with the smartphone’s screen recording feature, where it is located, how to start and stop recording and where the recording is saved on their phone. This gave them a sense of confidence and enabled them to complete the screen capture task in less than 5 min.

*‘Yeah, very easy because I’ve used the screen recording feature before, so I was very familiar where it is and how do I record, where it gets saved’.* (B10, phone group)

*‘I spent around – no more than two minutes because I’m very familiar with the recording thing’.* (B1, phone group)

For participants using the third-party screen recording app, most expressed a high level of satisfaction describing the entire experience of using the app as ‘straightforward’, from downloading the app to using it and uploading the recording on the app’s dashboard. While no participant had previously used the app, participants felt the instructions provided at the start of the task describing the process of using the app to screen record their purchase were very comprehensive and clear.

*‘I think it was a pretty easy process. It’s just screen recording and then you stop when you’re done so no issues with that’.* (C25, app group)

*‘It was all really straightforward’.* (C12, app group)

In addition to the overall ease of using the screen recording feature reported by most participants in both phone and app group, they also expressed the process of using the screen capture method to record their food selection and purchase on the OFD platform as feasible. Compared to the participants in the phone group, all participants using the third-party screen recording app described an enhanced ability to switch between the third-party screen recording app and the OFD service app to place an online food order due to step-by-step guidance that was provided on the third-party screen recording app.

*‘I feel like the interface and the instructions were clear and it was very easy to follow steps. From switching between apps [OFD service app and the third-party screen recording app] and to recording, trimming the video and then uploading it. There’s a tick, so I just tick it and then it gets uploaded. I found it all very useful’.* (C3, app group)

#### Overall barriers and facilitators to the use of screen capture methods

Participants reported some barriers and enablers in performing the task when using the screen capture methods. The main barrier for most participants in both groups was remembering to ‘turn on’ the recording at the time of making a food order using the OFD service. Participants, in the app group, attributed this forgetfulness to the unfamiliarity of the screen recording app and preferred leaving the tasks for the next OFD order.

*‘I keep forgetting to record when I am ordering as I am often quite hungry and, in a rush,’.* (B2, phone group)

*‘I’m like, oh, I forgot to screen record but it’s all me and nothing to do with you or the task. And then, yeah, but then, there’s always the next time’.* (C2, app group)

In both groups, over half of the participants reported concerns related to the protection and privacy of their data while performing the task. For example, when pop-up notifications were received on the phone while performing the task, participants felt unsure about whether to proceed with the task at that moment or leave it for the next OFD in order to avoid uploading a screen recording that included personal notifications. Less than half the participants in the app group indicated that they felt uncomfortable using the ‘new’ app for the task as they were more familiar with their smartphone screen recording function and were more inclined to use that instead. Further, the time taken for some participants to complete the task in the app group was longer than 10 min, excluding the setup time (app download and log in), which exceeded the allocated recording time for upload, resulting in multiple attempts. Participants perceived these difficulties as a burden associated with participating in the study due to the amount of effort and time spent to complete the task.

*‘There were a couple of times where I would get a message or something while it was screen recording and either I’d just forget and I’d like open my messages, or it - I guess it was just kind of like privacy reasons’.* (B3, phone group)

*‘There’s just a lot, as you know, in the media about the potential dangers of downloading apps’.* (C14, app group)

*‘Maybe 10–15 min. Because of course it’s a new app, you have to get used to like an app before you proceed with the thing’.* (C10, app group)

All participants in both groups reported that in case of any difficulties illustrated above. They referred to the plain language statement to read all the procedures put in place to ensure data protection and privacy or contacted the primary author for clarifications. Participants reflected that these approaches restored their confidence and facilitated them to complete the task.

*‘I think I had to reread the tasks a few times, because I wasn’t too sure. But then I think once I reread your emails and reread the app again, I understood everything and I then complete the task’* (C18, app group)

All the participants unanimously referred to the usefulness, clarity and simplicity of the instructions provided to complete the task, as a key enabler for them to use the screen recording app with ease.

*‘Loved the fact that you have the instructions uploaded on the app itself. So, I feel like the interface and the instructions were clear and it was very easy to use’.* (C21, app group)

### Users’ exposure to and engagement with marketing strategies on online food delivery platform

All participants were exposed to, and engaged with, a range of marketing strategies on the OFD platform in multiple ways to influence their final purchase (Table 3). Adapting the marketing mix framework applied in the context of online grocery^(35,39)^, below we describe the marketing strategies employed by the OFD services on their platforms as observed by the screen recording data of OFD orders.

**Table 3.** Description, prevalence, location and examples of food and beverage marketing strategies used by online food delivery platforms (*n* 20 screen recordings)

Marketing strategy	Description	Homepage		Food outlet page		At checkout		Examples from the screen recording
		*n*	%	*n*	%	*n*	%	
Product								
Product mix	An assortment of food products recommended by the OFDS	14	70	20	100	19	90	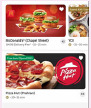
Filter option	Option to view products with specific characteristics	20	100	20	100	N/A		
Placement								
Best match	Best match or most relevant items are shown first by default	20	100	20	100	N/A		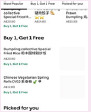
Featured	Featured items are shown first by default	20	100	20	100	N/A		
Most Popular	Most popular items are shown first by default	20	100	20	100	N/A		
Price								
Discounts	Discounts that can be redeemed at checkout	20	100	20	100	20	100	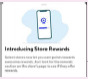
Loyalty rewards	Accrual of rewards that can be redeemed for savings	20	100	20	100	20	100	
Time-limited deals	Discounts of a limited duration	20	100	20	100	20	100	
Promotion								
Title cards	Large panel of imagery and text at the top of the page featuring foods or beverages	18	90	N/A		N/A		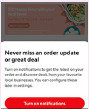
Banner advertisements	Rectangular Internet display advertising format driving visitors to a post-click landing page, featuring food or beverages	20	100	N/A		N/A		
Push notification	In app alert (typically a pop-up or other message)	10	50	N/A		N/A		
Member special offers	Savings only for loyalty program members	5	25	N/A		N/A		

OFD, online food delivery.

#### Product strategy

Overall, the assortment of a range of (less healthy) food items on the homepage (*n* (%) = 14 (70 %)), food outlet page (20 (100 %)) and checkout page (18 (90 %)) of the OFD platform appeared similar for over half the participants. For a small number of participants (*n* 5), the assortment of food offerings that appeared on their homepage, food outlet page and checkout, closely matched with their previously ordered food items (past purchases). Filter options were available on both the homepage (20 (100 %)) and the food outlet page (20 (100 %)) to view food products with specific characteristics. Participants often navigated through the recommended assortment of food items and filter options to search for food products to inform their decision-making process.

#### Placement strategy

A range of fast-food outlets offering a variety of food items appeared on the first half of the homepage (20 (100 %)), just before the independently operated food outlets (20 (100 %)). The food offerings were mostly tagged as recommended or best match (20 (100 %)) and were found to align with participants’ purchase history of previous orders (*n* 14 (70 %)) on the same OFD platform. For instance, a participant who had ordered a meal from a nearby fast-food restaurant days prior to the recording task saw similar meals from a range of other fast-food outlets as the first image of the food item on opening the OFD service app. When participants clicked on these fast-food outlets, similar meals were tagged as featured items and most popular items and appeared as default items to inform food purchase decisions.

#### Price strategy

Within the price strategy, a predominant presence of discounts (20 (100 %)), loyalty rewards (20 (100 %)) and time-limited deals (20 (100 %)) was observed in the screen recordings. The food outlets on the OFD platform, for example, highlighted ‘customer favourites’ (aligned with previous purchase history) with discount offers such as ‘spend $35[AUD] [USD 19] and save $10[USD7]’, ‘Buy1, Get 1 free’ and ‘save $10[USD7]’; ‘deals under $30AUD [19USD]’; ‘free item (when you spend over $30AUD [19USD)’; loyalty rewards such as ‘two orders away before $25 [USD16] off’ and time-limited deals such ‘15 % off your order today’ or ‘Order now and save up to $30 [USD19]’. Additionally, ‘low/$0 delivery fee’ was another prominent feature commonly observed for the premium members of the OFD service in the screen recordings. All participants actively searched for discounts and offers on new dishes, past favourite food items from specific restaurants, combo/meal deals or time-limited promotions, which informed their decision-making process.

#### Promotion strategy

Participants were exposed to a range of promotional marketing strategies on the homepage from running title cards (18 (90 %)) and banner advertisements (20 (100 %)) (such as ‘Get almost, almost anything’ or ‘hot deals from your local faves (sic)’ or ‘in a rush?’ to showcasing high-quality and visually appealing images of food items and identify them as ‘most liked’ (signposted with a thumb up), ‘most rated’ (signposted with a medal or a star) and ‘short delivery time’. Half of the participants (with or without premium memberships) received in-app targeted push notifications on the homepage (10 (50 %)), to cross-promote featured food products in the form of multi-buys, such as ‘try something new for less’; ‘suggested complementary products’ based on their preferences and purchase history (as confirmed by their past orders). Other examples of *promotion strategies* that were frequently seen were ‘order again’, ‘our picks for you’ and ‘sad empty fridge?’ (for grocery delivery). Participants with premium memberships were prompted on ‘$0 delivery fee’ (5 (25 %)) for almost all restaurants available on the OFD platform. Participants without premium memberships were seen to receive in-app targeted push notifications nudging them to acquire a membership to receive ‘$0 Delivery Fee and reduced Service Fee (up to 30 % off)’ or ‘receive $5 credit on late order arrivals’.

#### Engagement

All the above-described *marketing strategies* were frequently clicked and were applied in combination with each other, to influence search and selection on the homepage and the food outlet page, and most commonly during purchase at the checkout page on the OFD platform. Participants with no premium memberships often spent extra time (nearly 1 min) to review their selected items and re-engage with multiple *price and promotion strategies* (such as ‘no delivery fee’, ‘15 % off your order today’, ‘applying a promotional code or discount voucher’) to reduce their overall cost of the food order (including service and delivery fee). For example, at checkout, one participant switched from a discounted food item at a popular outlet over 3 km away to the same item signposted as a *‘suggested for you’,* from a closer outlet (< 1 km away) that offered both a discount and free delivery. However, participants with premium memberships selected products to meet the minimum cost of the order criteria to qualify for free delivery and reduced (or zero) service fee.

## Discussion

This study aimed to assess the consumer acceptability of two screen recording methods for capturing their real-time engagement with OFD service and describe users’ exposure to, and engagement with, marketing strategies used on the OFD platform. Our findings suggest that both screen recording methods were well accepted by participants and are a feasible approach to capture the dynamic user experience on the OFD platform. The participants took on average less than 5 min to complete the recording which was between 2 and 5 min. As researchers, the data analysis took less than 20 min to code one video according to the 4Ps of marketing strategies, establishing the possibility of replicating the study with a large sample size. A range of marketing strategies were visible on the homepage of the OFD platform, food outlet page and on checkout page on the OFD platform. These included the presence of a mix of food products, strategic placement of selected food products, price discounts and promotion strategies. Participants engaged with these marketing strategies through multiple clicks to reduce the total cost of their OFD order.

Screen recording has been largely used in the field of information technology, education and communication^(40)^, for the purpose of research data collection. Screen recording allows for a holistic observation of user behaviours, providing a nuanced understanding of the decision-making process in real-time, navigation patterns and temporal aspects of online engagement^(41)^. This aligns with recent research emphasising the importance of real-time data capture in the digital realm^(22,29,41–43)^ to comprehensively map user’s actual exposure to, and engagement with, online marketing strategies. In our study, all participants expressed a high level of user satisfaction in applying the screen recording methods to capture their food purchase journey using an OFD service. In addition to the consumer perspective, from the researcher’s perspective, the screen capture data provided us with a rich insight into the user’s exposure to the expanding volume, variety and dynamism of marketing practices on the OFD platform. Our study supports the existing evidence that suggests that screen recording is an effective method for real-time data observations^(41)^. While most participants expressed overall satisfaction with the usefulness, clarity and simplicity of the detailed instructions on the process, there were some concerns raised by participants on the privacy of their personal data (e.g. chats, emails, banking) being recorded while undertaking the task. These concerns were similar to those observed in previous studies^(44,45)^ and addressed by our research team by reiterating the data management procedure (i.e. all participants were reminded to turn off all notifications before commencing the food p[purchase task and that participant data are stored in a secure password protected file in the University Research Data Store) to the participants. Future research could explore innovative ways (e.g. developing browser extensions^(46)^) to automatically stop notifications from popping up once the screen recording app is turned on. This could effectively enhance the benefits of such methods for research-related purposes and reduce participant’s perceived data privacy concerns. Screen recording methods hold great potential for research to understand consumer exposure and engagement with marketing techniques when making food choice decisions on the OFD platforms. Our study also provides insights into user’s acceptability of screen recording tools to study the process of food selection and purchasing on OFD platforms. Future research evaluating the relationship between the user acceptability and cost of using screen recording tools to ensure that they are effective tools for both participants and researchers, respectively, will be valuable. Future research could use the screen recording methods to investigate how diverse population sub-groups interact with the marketing strategies on OFD platforms in real time.

A wide range of marketing strategies both individually and in combination were observed across the homepage, food outlet page and at checkout on the OFD platform. These included a presence of a mix of recommended food products on the different pages, strategic placement of selected food products that appeared as default on homepage and food outlet page; price discounts and promotion strategies to increase the visibility of a product across all pages. Multiple forms of price strategies combined with promotion on food products were frequently observed across the homepage, food outlet page and checkout page on the OFD platform. Previous studies suggest that marketing strategies employed by other online food retail platforms such as online grocery retail are primarily to enhance user experience and increase their purchase intention^(39)^. This was clearly observed in the screen recordings where the participants in our study were seen to engage with these strategies at multiple points in their purchase journey to influence the final cost of the food order. Furthermore, there is emerging evidence suggesting that these pervasive marketing strategies on the OFD platforms are becoming increasingly personalised and targeted based on race, ethnicity or socio-economic status, previous browsing and purchase history^(47)^. There is also evidence that these strategies promote a high proportion of nutrient-poor food^(48)^ and increased accessibility of OFD service in low socio-economic areas^(49)^ affecting vulnerable population groups with adverse health outcomes disproportionately. While we could not assess in-depth to what extent the marketing strategies employed by the OFD platforms on their platforms were personalised or targeted in our study, there were some indications on the alignment of the marketing strategies with selected food products and participants purchase history, which were mostly energy-dense food items. Future studies with larger sample sizes are needed to better understand the impact of continuously evolving forms of marketing strategies (personalised and targeted) on food purchase behaviour, for different population sub-groups, on OFD platforms. This will help determine the inequity in food purchased by consumers using the OFD platform and inform solutions with equity considerations to mitigate the impacts of diet on chronic diseases.

Previous studies have shown that consumers use OFD platforms for various reasons such as convenience, cost savings and others^(36)^. When these consumer-related factors combine with the pervasive marketing strategies promoting a high proportion of nutrient-poor food on the OFD platforms^(14,50)^, it is likely to increase the purchases of these foods on OFD platforms. This may eventually result in overconsumption of unhealthy food^(51)^. As the global trends in OFD platform use are rising^(7)^, our findings highlight an urgent need to design and implement interventions or public policies aimed at mitigating consumer’s exposure to the marketing of unhealthy food and encouraging healthy food purchases on the OFD service^(52)^. OFD platforms could restructure its digital interface to nudge consumers towards healthier food purchases by altering the assortment of food products to display healthier food items as the default, display promotions on healthy food items on the menu and offer recommendations on healthy food swaps that meet the Australian Dietary Guidelines^(53)^ for healthy food. Food outlets should be required to increase the proportion of healthy food items available and potentially expand their food labelling practices including both in-store and online food retail outlets^(54)^. Whilst evidence on the public health impact of OFD platforms is limited, replacing marketing strategies that promote unhealthy products with marketing for healthier products is likely to have positive population health impacts. However, as OFD platforms are a profit-driven entity, pivoting to promoting healthy products may put them at a competitive disadvantage^(55)^. Hence, government-led policies are critical to create a level playing field for the OFD platforms. Continuous monitoring of the exposure to the pervasive marketing strategies on the OFD platforms is needed to demonstrate the public health impact of OFD platforms. This will also provide an impetus for the need for government-led actions to regulate and restrict the ways in which consumer data are exploited to personalise marketing strategies on the OFD platforms^(56)^.

### Strengths and limitations

This is the first study to demonstrate that screen recording is an acceptable method to capture consumer’s real-time exposure and engagement with food marketing strategies on the OFD platforms. We captured participants’ ‘actual’ exposure to the digital marketing strategies applied on OFD platforms across the entire purchase including homepage, within the food outlets menu page until the checkout. This study provides primary evidence of an emerging yet understudied type of online food retail setting i.e. OFD platforms. Last, as the primary author of this study is a public health researcher and has previously used OFD platforms, caution was taken to minimise researcher bias by documenting thoughts, assumptions and personal biases during the process of the research study. However, through reflexivity, we acknowledge that our analysis may be influenced by our public health research knowledge and our experience with the use of OFD platforms. There are some limitations to this study that must be considered when making inferences. The demographics of our study population i.e. mostly female adults with a high level of education, living in major cities, may not be representative of the most common users of OFD platforms (mostly young and men^(36)^) in Australia and globally, thereby limiting the generalisability of our results. Further, this is a study with a small sample size, recruited using the university social media accounts, and therefore, this sample is likely to have high digital literacy. Thus, future research is needed to establish the credibility of our findings on a large and diverse sample size. Our findings indicated some level of participant burden (related to the protection and privacy of their data) of using screen recording methods and very limited on the researcher’s burden (time spent) for coding data generated using screen recording methods. More data from future studies are needed to fully understand both the participant’s and researcher’s burden of using the screen recording methods. Next, we were limited in our ability to comment on whether the marketing strategies applied to selected food products were personalised or targeted. Future studies with larger sample sizes could assess whether and how marketing strategies are applied differently for healthy and unhealthy food products on the OFD platforms. Furthermore, future studies are needed to ascertain the extent to which the marketing strategies on selected food products may influence consumer’s purchase of the selected food products. Last, while limited, in our study, user engagement with marketing strategies on OFD platforms was assessed based on clicking on the marketing strategies to influence search, selection and purchase. Future studies should employ a combination of methods such as eye tracking methods^(21)^ and follow-up qualitative inquiries to better capture user engagement with marketing strategies on the OFD platform.

### Conclusion

This study identified screen recording as a feasible method for capturing consumers’ real-time exposure and engagement with marketing strategies on the OFD platform. Using screen recordings, our study highlighted that Australian adult users are exposed to a wide range of digital marketing strategies on the homepage of the OFD platform, the food outlet page and the checkout page on the OFD platform. These included the presence of, and strategic placement of selected food products, price discounts and promotion strategies on the OFD platforms. More understanding of how these marketing strategies are personalised and targeted to influence consumer’s actual purchase behaviour on OFD platforms is needed.

## Supporting information

Gupta et al. supplementary material Gupta et al. supplementary material

## Supplementary material

For supplementary material accompanying this paper visit https://doi.org/10.1017/S1368980025000515
