# Supplementary material for: Consumers acceptability of using screen capture methods to capture marketing strategies on online food delivery platforms: a qualitative study
Source: Public Health Nutr. 2025 Apr 14;28(1):e88. doi: 10.1017/S1368980025000515 (PMC12100556; doi:10.1017/S1368980025000515)
Supplement: Gupta et al. supplementary material [file S1368980025000515sup001.docx]

Supplementary Table 1: Semi-structured interview guide

| LOGIC | QUESTIONS | *PROBES* |
| --- | --- | --- |
| Reflection on the usability of the two screen capture methods used | - Describe your experience of   a) clicking/ recording features that you used within the phone/ indeemo app  b) features that you used within the phone/ indeemo app to capture the screen recording of the food | - What do you think about this method? - What do you think about the ease and efficiency of this method? - How much time did you spent in completing the task (*usability/efficiency*)? What does it mean for you? Why do you think it took you that much time? - Did you make some kind of errors? What were they and how many times you think you made errors? Why do you think you made them? - If yes- what do you think about the second or third time using this feature? Was it any different to you first attempt? What led you to do it differently? Were you able to remember the steps? Why or why not? |
| Reflection on the acceptability of the two screen capture methods used | - Describe how you felt during and after completing the task and uploading the file (video recordings)? | - Did the way the information provided to you on how to use phone/ indeemo to record your purchase useful/ not useful? (From opening the app, identifying the task, understanding the interface, navigation features to completing the task- *usability*) - Tell me how and why that was useful/ not useful? - What are your views about the support offered in the form of instructions to complete the task (*effectiveness*)? - Did you feel satisfied about the entire process? - What do you think was missing? - How do you think it impacted your ability to complete the task? |
| Overall reflection on the user satisfaction of the two screen capture methods used | - What do you think about the overall facilitators (helpful) and barriers (challenging) of the method used? | - What do you think are the things that you felt were helpful in the entire process? - What do you think are the things that you felt were challenging and limited you from completing the task? |
